# All trans-retinoic acid (ATRA) induces re-differentiation of early transformed breast epithelial cells

**DOI:** 10.3892/ijo.2014.2354

**Published:** 2014-03-21

**Authors:** MARIA F. ARISI, REBECCA A. STARKER, SANKAR ADDYA, YONG HUANG, SANDRA V. FERNANDEZ

**Affiliations:** 1 Kimmel Cancer Center, Thomas Jefferson University, Philadelphia, PA 19107;; 2 Section of Gastroenterology, Department of Medicine, University of Chicago, Chicago, IL 60637, USA

**Keywords:** breast cancer, vitamin A, retinoic acid, transformation, branching

## Abstract

Retinoids have been used as potential chemotherapeutic or chemopreventive agents because of their differentiative, anti-proliferative, pro-apoptotic and antioxidant properties. We investigated the effect of all trans-retinoic acid (ATRA) at different stages of the neoplastic transformation using an *in vitro* model of breast cancer progression. This model was previously developed by treating the MCF-10F human normal breast epithelial cells with high dose of estradiol and consists of four cell lines which show a progressive neoplastic transformation: MCF-10F, normal stage; trMCF, transformed MCF-10F; bsMCF, invasive stage; and caMCF, tumorigenic stage. In 3D cultures, MCF-10F cells form tubules resembling the structures in the normal mammary gland. After treatment with estradiol, these cells formed tubules and spherical masses which are indicative of transformation. Cells that only formed spherical masses in collagen were isolated (trMCF clone 11) and treated with ATRA. After treatment with 10 or 1 *μ*M ATRA, the trMCF clone 11 cells showed tubules in collagen; 10 and 43% of the structures were tubules in cells treated with 10 and 1 *μ*M ATRA, respectively. Gene expression studies showed that 207 genes upregulated in transformed trMCF clone 11 cells were downregulated after 1 *μ*M ATRA treatment to levels comparable to those found in the normal breast epithelial cells MCF-10F. Furthermore, 236 genes that were downregulated in trMCF clone 11 were upregulated after 1 *μ*M ATRA treatment to similar levels shown in normal epithelial cells. These 443 genes defined a signature of the ATRA re-programming effect. Our results showed that 1 *μ*M ATRA was able to re-differentiate transformed cells at early stages of the neoplastic process and antagonistically regulate breast cancer associated genes. The invasive and tumorigenic cells did not show any changes in morphology after ATRA treatment. These results suggest that ATRA could be used as a chemopreventive agent to inhibit the progression of premalignant lesions of the breast.

## Introduction

Vitamin A is obtained through the diet in the form of retinol, retinyl ester or β-carotene (1). Retinoic acid (RA) is one of the principal active metabolites of vitamin A which plays a critical role in cell proliferation, differentiation and apoptosis in normal tissues during embryonic development (2). RA induces differentiation in many cell types and is the most widely used differentiating therapeutic agent (3,4). Retinol has 6 biologically active isoforms that among others includes all-trans (ATRA, tretinoin) and 9-cis RA (alitretinoin); ATRA is the predominant physiological form (5). RA mediates the transcriptional regulation of several genes by binding to the nuclear retinoic acid receptors (RARs), namely RARα, RARβ and RARγ (6,7). Like other nuclear receptors, RARs contain a domain that mediates interaction with ATRA, a zinc finger-containing DNA binding domain that binds to RA response elements (RAREs) in target genes, and a dimerization domain that engages members of the retinoid X receptor (RXR) subfamily in RXR/RAR heterodimers (8). Different isomers activate different receptors and thus lead to different biological effects. RARs can be activated by both all-trans (ATRA) and 9-cis-RA, while RXR are exclusively activated by 9-cis RA; however, due to the conversion of ATRA to 9-cis RA, high concentrations (10^−5^ M) of ATRA can also activate gene transcription in cells transfected with RXRs (9). It has also been shown that retinoids exert their effects via the nuclear receptor independent pathway (5).

RA and its derivatives are promising anti-neoplastic agents endowed with both therapeutic and chemopreventive potential because they are able to regulate cell growth, differentiation and apoptosis (10,11). It is believed that the anti-neoplastic pathways induced by RA are regulated predominantly by RAR-β, which is known to induce apoptosis; thus it has been suggested that RAR-β plays a critical role in mediating the growth arrest and differentiation in several breast cancer cell types (12–14).

**Figure 1.**
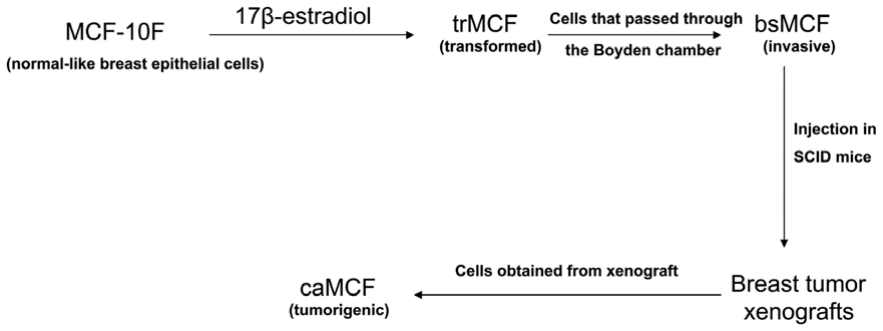
*In vitro*-*in vivo* model of cell transformation. The human normal-like MCF-10F cells were treated with high dose of estradiol and named early transformed breast epithelial cells (trMCF). The trMCF cells were seeded on a Boyden chamber and the cells that invaded, bsMCF, were selected and expanded. The bsMCF cells were injected in the fat mammary pad of SCID mice producing breast tumor xenografts. These xenografts were surgically removed and tumor cells were expanded giving origin to caMCF. The trMCF cells did not produced tumors when injected in SCID mice.

We have developed an *in vitro*-*in vivo* model of breast cancer progression by treating the human normal-like breast epithelial cells MCF-10F with a high dose of estradiol (70 nM) (Fig. 1) (15,16). This model consists of four cell lines: i) the spontaneously immortalized cell line MCF-10F, which is considered to be a normal-like breast epithelial cell line; ii) the transformed trMCF cells; iii) the invasive bsMCF cells; and iv) cells isolated from xenografts, caMCFs, which show all characteristics of fully malignant breast cancer cells (Fig. 1). Gene expression studies showed the highest number of deregulated genes in caMCF, being slightly lower in bsMCF, and lowest in trMCF and, this order was consistent with the extent of chromosome aberrations (caMCF>bsMCF>>trMCF) (16). This model of breast cancer progression resembles the different steps of neoplastic transformation of the mammary gland; it is widely held that breast cancer initiates as the premalignant stage of atypical ductal hyperplasia (ADH), progresses into the pre-invasive stage of ductal carcinoma *in situ* (DCIS), and culminates in the potentially lethal stage of invasive ductal carcinoma (IDC) (17). In collagen, the normal-like MCF-10F cells form tubules resembling the structures observed in the normal mammary gland although after treatment with estradiol, the transformed trMCF cells form tubules and spherical masses, which are indicative of cell transformation (8,19). The spherical masses showed a partial filling of the lumen that would result from decreased central apoptosis, enhanced cellular proliferation or both (18). The filling of the lumen of the tubular structures of the breast is the earliest morphologic alteration and is common in atypical ductal hyperplasia and ductal carcinoma *in situ* (DCIS) (18). In the presented study, we studied the effect of all trans-RA (ATRA) using this model of breast cancer progression. Our results showed that ATRA was able to re-program early transformed cells to a normal stage.

## Materials and methods

### Cells and media

The human normal-like breast epithelial cells MCF-10F are estrogen receptor (ER) negative, progesterone receptor (PR) negative and HER2 negative. Cells were cultured in Dulbecco’s modified Eagle’s medium [DMEM/F-12, Gibco, Carlsbad, CA; formula 90–5212 EF: containing DMEM/F12 (1:1) with L-glutamine and phenol red, D-glucose 315 mg/l, sodium pyruvate 55 mg/l] with 5% horse serum, 2.43 g/l sodium bicarbonate, 20 mg/l epidermal growth factor (EGF), 100 mg/l *Vibrio cholerae* toxin, 10 mg/l insulin, 0.5 mg/l hydrocortisone, 1.05 mM calcium, antibiotics and antimicotic (100 U/ml penicillin, 100 mg/ml streptomycin, 0.25 mg/ml amphotericin). A 10-mM solution of all trans-retinoic acid (ATRA, Cat# R2625, Sigma, St. Louis, MO) was prepared as a stock solution by dissolving ATRA in dimethylsulphoxide (DMSO).

The trMCF clone 11 was isolated by seeding 100–1,000 trMCF cells in a 100-mm cell culture plate and after 1 day in culture, several colonies were isolated by ring cloning. The trMCF clone 11 cells were generated by expanding the cells from one of these colonies; trMCF clone 11 cells only formed spherical masses on collagen. To study the effect of ATRA, trMCF clone 11 cells were treated continuously for 26 days with 10^−5^ M (10 *μ*M) to 10^−8^ M (0.01 *μ*M) ATRA (media was replaced daily). As control, the cells were treated with 0.1% DMSO (vehicle). The bsMCF and caMCF cells were treated with 10^−5^ to 10^−8^ M ATRA alone or in combination with 2.5 *μ*M 5-aza-dC.

### Collagen assays

The cells were resuspended at a final density of 1.5×10^4^ cells/ml in collagen matrix consisting of 2.68 mg/ml (89.3%) type I collagen (PureCol, Inamed Biomaterials Co., Fremont, CA), 8% 12.5X DMEM-F12 with antibiotics, 0.1 mg/ml insulin, 14 mM NaHCO_3_ and 0.01 N NaOH. A total of 400 *μ*l (3,000 cells) were plated on the top four 24-well chambers pre-coated with 400 *μ*l of 89.3% collagen mix. Per each treatment, cells were plated in 4 wells and fed daily with the medium described before. The structures in collagen matrix were observed daily under an inverted microscope and at the end of the observation period (8 days), the structures (spherical masses, tubules and intermediate structures) were counted, photographed and fixed in 10% neutral buffered formalin and processed for histological examination. Results were expressed as the total number of structures per well (spherical masses, tubules and intermediate structures) and percentage of the different structures per treatment. The t-test was used to determine if the differences were significant.

### Invasion assays

Cell invasion in real-time were performed using xCELLigence RTCA DP device from Roche Diagnostics (Mannheim, Germany). For this purpose, each well of the upper chamber of the CIM-Plate 16 was covered with Matrigel (BD Biosciences, Franklin Lakes, NJ) basement membrane matrix (1:20 in cell culture media) and 10% fetal bovine serum (chemo-attractant) was added in the lower chamber. A total of 40,000 cells suspended in 100 *μ*l serum free media were seeded per well in CIM-Plates 16 (Roche Diagnostics). Data acquisition and analysis was performed with the RTCA software (version 1.2, Roche Diagnostics). Changes in impedance from cells that invade and migrate to the underside of wells were recorded and monitored for a total of 24 h.

### Gene expression profiling

RNA was isolated from the cells using RiboPure™ kit (Life Technologies, Frederick, MD) and RNA quality was controlled using the Agilent 2100 Bioanalyzer. Gene expression studies were performed using Affymetrix U133 Plus 2.0 (Affymetrix, Santa Clara, CA) human oligonucleotide microarrays containing over 47,000 transcripts and variants, including 38,500 well characterized human genes. After hybridization, the chips were scanned using GeneChip Scanner 3000. The data were analyzed with Microarray Suite version 5.0 (MAS 5.0) using Affymetrix default analysis settings and global scaling as normalization method. The trimmed mean target intensity of each array was arbitrarily set to 100. Background correction and normalization was done using Iterative Plier 16 with GeneSpring V11.5 software (Agilent, Palo Alto, CA). The criteria for differentially expressed genes was set at ≥2-fold changes (p-value <0.05). The differentially expressed gene list was loaded into Ingenuity Pathway Analysis (IPA) 8.0 software (Ingenuity Systems, Redwood City, CA) to perform biological network and functional analyses.

## Results

### Treatment with ATRA induced branching morphogenesis in early transformed breast epithelial cells

**Figure 2.**
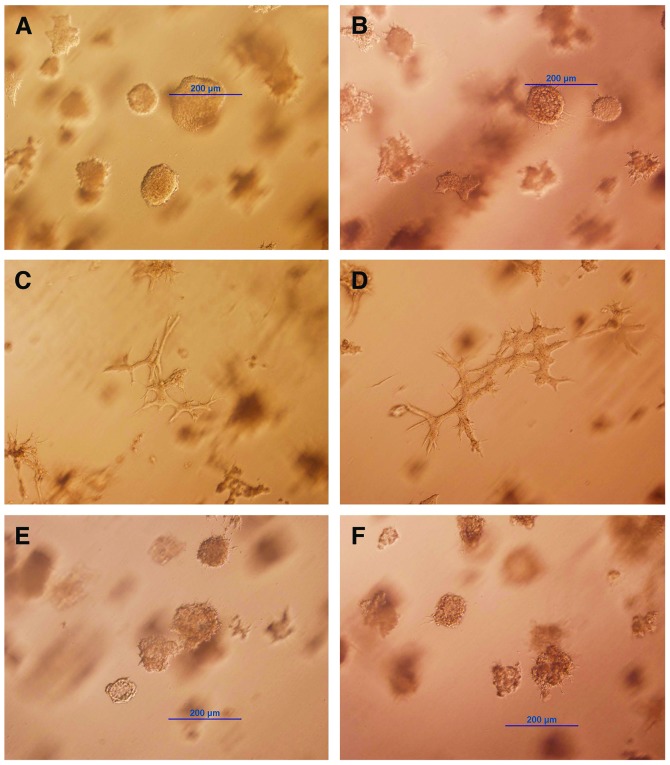
All-trans retinoic acid (ATRA) induces morphological changes in transformed cells trMCF clone 11. The trMCF clone 11 cells were plated in collagen matrix (3-D cultures) after being treated continuously for 26 days with different concentrations of ATRA. The cells were photograph after 8 days in collagen. (A) trMCF clone 11 cells (control); (B) trMCF clone 11 cells treated with 0.1% DMSO (vehicle, control); (C) trMCF clone 11 after being treated with 10^−5^ M (10 *μ*M) ATRA; (D) trMCF clone 11 cells after being treated with 10^−6^ M (1 *μ*M) ATRA; (E) trMCF clone 11 cells after being treated with 10^−7^ M ATRA; (F) trMCF clone 11 cells after being treated with 10^−8^ M ATRA.

**Figure 3.**
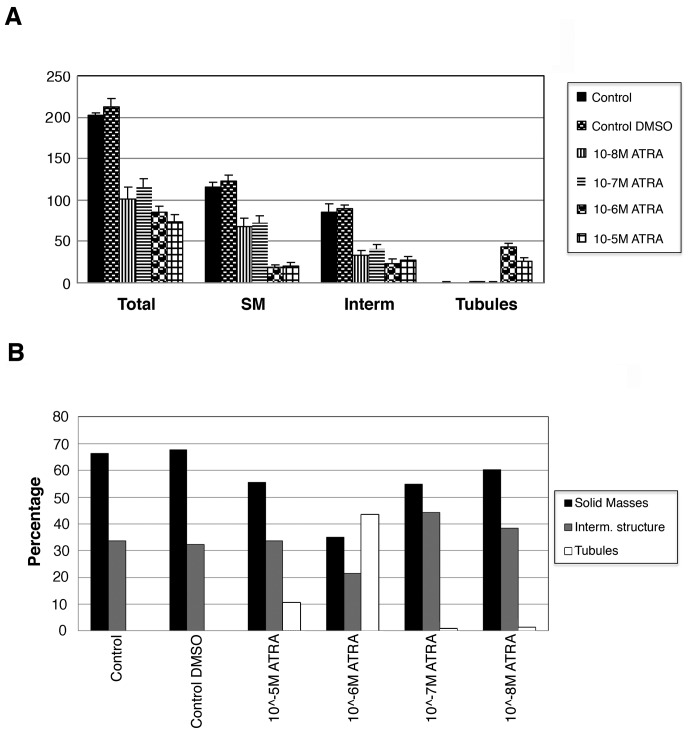
Spherical masses, tubules and intermediate structures formed in collagen by trMCF clone 11 before and after ATRA treatments. The trMCF clone 11 cells form spherical masses in collagen and some intermediate structures. The trMCF clone 11 cells were treated with different concentrations of all-trans retinoic acid (ATRA) for 26 days; after ATRA treatments, the cells were plated in collagen. (A) Total number of different structures in collagen of trMCF clone 11 cells before and after ATRA treatment. Total number of spherical masses (SM), tubules and intermediate structures (spherical masses with prolongations) per well are shown. (B) Different structures on collagen matrix of trMCF clone 11 cells after treatment with different concetrations of ATRA. Percentage of different structures in collagen.

MCF-10F cells are normal-like breast epithelial cells that form tubules in collagen matrix (3D culture); when these cells were treated with high dose of estradiol (70 nM), the cells (trMCF) formed tubules and spherical masses. To isolate transformed cells that only form spherical masses, trMCF cells were seeded at low density in cell culture dishes and several clones were isolated by ring cloning. One of these clones, trMCF clone 11, did not form tubules in collagen; instead these cells formed spherical masses and intermediate structures (Fig. 2A and B). The trMCF clone 11 cells were treated continuously for 26 days with 10^−5^ to 10^−8^ M all trans-retinoic acid (ATRA) and, we found that cells treated with 10^−5^ and 10^−6^ M ATRA were able to form tubules in collagen (Fig. 2C and D). Furthermore, the spherical masses formed by trMCF clone 11 treated with 10^−5^ and 10^−6^ M ATRA (Fig. 2C and D) were smaller compared to the ones formed by the controls (Fig. 2A and B) or cells treated with 10^−7^ and 10^−8^ M ATRA (Fig. 2E and F). The trMCF clone 11 cells treated with 10^−7^ and 10^−8^ M ATRA (Fig. 2E and F) did not show any difference in morphology when compared to the controls (Fig. 2A and B). The number of spherical masses, intermediate structures and tubules for trMCF clone 11 cells treated with different concentrations of ATRA was counted (Fig. 3). The total number of structures in collagen was significantly lower in cells treated with ATRA compared with the controls suggesting that ATRA treatment decrease the proliferation rate of the cells (p<0.01) (Fig. 3A). The control trMCF clone 11 showed spherical masses and intermediate structures but no tubules in collagen while cells treated with 10^−6^ and 10^−5^ M ATRA formed tubules and less spherical masses (Fig. 3A). The cells treated with 10^−5^ or 10^−6^ M ATRA formed significantly less spherical masses than the cells treated with 10^−7^ or 10^−8^ M ATRA (p<0.01) (Fig. 3A). A total of 43% of the structures were tubules in the wells containing cells treated with 10^−6^ M ATRA and 10% tubules in wells with 10^−5^ M ATRA-treated cells (Fig. 3B).

**Figure 4.**
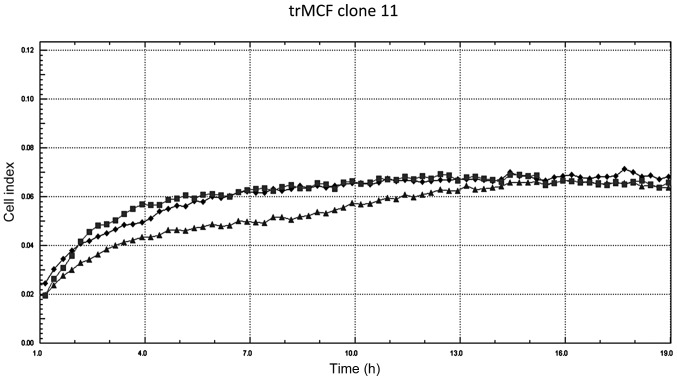
Invasion assay of trMCF clone 11 before and after ATRA treatments. The cell index of Matrigel-coated wells (invasion) at different time points are shown. The invasion capacity of the trMCF clone 11 did not show significant differences after 10^−5^ M ATRA (▲) or 10^−6^ M ATRA (■) compared with control after 16 h.

The invasion capacity of trMCF clone 11 was studied before and after ATRA treatment but, no differences were observed (Fig. 4). The bsMCF and caMCF cells did not show any changes in their morphology or invasion capacity after treatment with ATRA alone or in combination with the demethylating agent 5-aza-cytidine (data not shown).

### Treatment with ATRA re-programmed gene expression of early transformed cells

**Figure 5.**
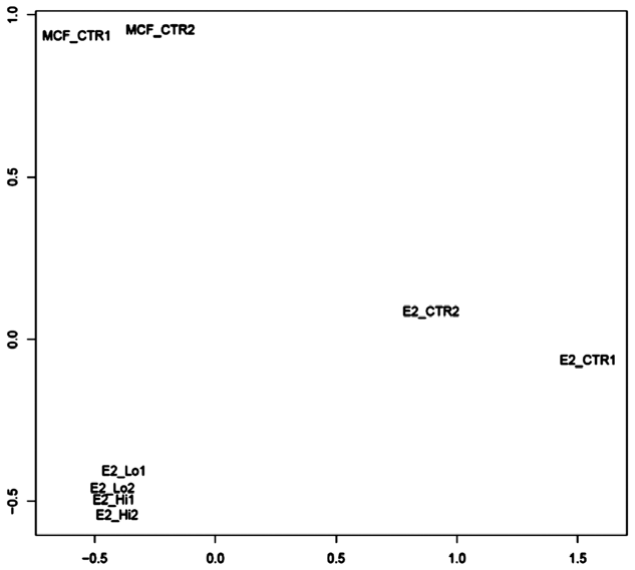
Unsupervised sample classification by principle coordinate analysis (PCoA). Two arrays were performed for each cell line and treatment: MCF-10F (MCF_CTR1 and MCF_CTR2), trMCF clone 11 (E2_CTR1 and E2_CTR2), 10^−5^ M ATRA trMCF clone 11 (E2_Hi1 and E2_Hi2) and 10^−6^ M ATRA trMCF clone 11 (E2_Lo1 and E2_Lo2). The trMCF clone 11 cells treated with 10^−5^ M ATRA (E2_Hi) or 10^−6^ M ATRA (E2_Lo) shown a major difference with trMCF clone 11 cells (E2_CTR) and minor differences with MCF-10F cells (MCF_CTR).

**Figure 6.**
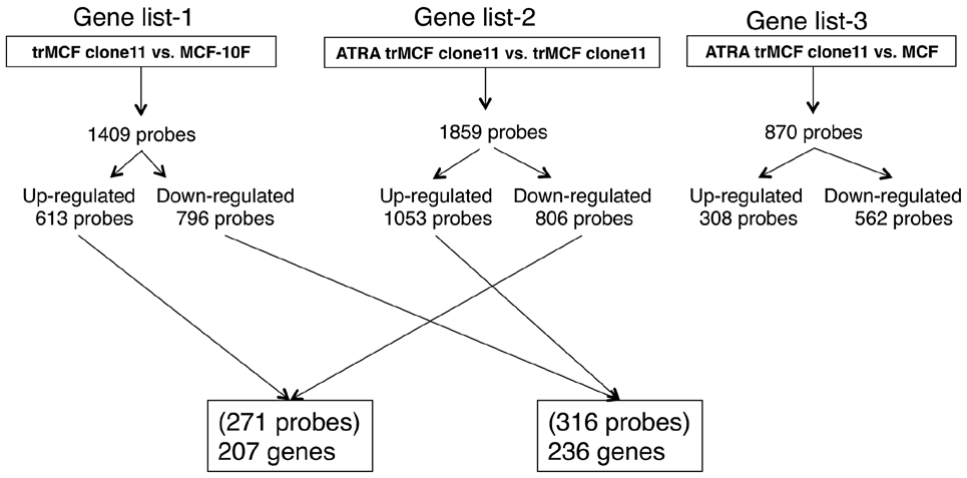
Representation of the gene expression studies showing number of dysregulated genes. Expression studies were performed in the early transformed trMCF clone 11 cells before and after treatment with 10^−6^ M ATRA (ATRA trMCF clone 11) and normal human breast epithelial MCF-10F cells. A total of 207 genes upregulated in the transformed trMCF clone 11 (compared to the normal MCF-10F) were downregulated after treatment with 10^−6^ M ATRA and, 236 genes that were downregulated in trMCF clone 11 (compared to MCF-10F) were upregulated by 10^−6^ M ATRA treatment. These 443 genes defined a gene signature programming the reverse-transformation effect by ATRA.

As trMCF clone 11 cells that only formed spherical masses on collagen were able to form tubules after treatment with 10^−5^ or 10^−6^ M ATRA, gene expression studies were performed on these cells. The microarray data have been deposited into the NCBI gene expression omnibus (GEO) datasets (GSE51549). The unsupervised sample classification by PCoA (principle coordinate analysis) revealed that trMCF clone 11 cells treated with 10^−5^ or 10^−6^ M ATRA demonstrated a major difference with trMCF clone 11 cells, and a minor difference with MCF-10F; also sample differences between 10^−5^ M ATRA and 10^−6^ M ATRA were weak (Fig. 5). Although, trMCF clone 11 cells treated with 10^−5^ M ATRA and 10^−6^ M ATRA showed minor differences at the expression level, we considered trMCF clone 11 treated with 10^−6^ M ATRA for the expression analysis since the number of tubules in collagen matrix was higher for this concentration (43% tubules with 10^−6^ M ATRA vs. 10% tubules with 10^−5^ M ATRA). For gene expression studies, three experimental groups were compared using empirical Bayesian-moderated t-test implemented in R package ‘limma’: the normal breast epithelial cells MCF-10F, the cells transformed by treatment with estradiol trMCF clone 11 (that only formed spherical masses on collagen) and the trMCF clone 11 after treatment with 10^−6^ M ATRA. We generated three gene lists at criteria of fold change ≥2 and p≤0.05: gene list-1 (trMCF clone11 vs. MCF-10F) with 1,409 probes (613 probes upregulated; 796 probes downregulated), gene list-2 (ATRA trMCF clone 11 vs. trMCF clone 11) with 1,859 probe sets (1,053 probes upregulated; 806 probes downregulated) and gene list-3 (ATRA trMCF clone 11 vs. MCF-10F) with 870 probe sets (308 probes upregulated; 562 probes downregulated) (Fig. 6). Most importantly, 207 genes (271 probes) upregulated in the transformed trMCF clone 11 (compared to the normal MCF-10F) were downregulated after treatment with 10^−6^ M ATRA (Fig. 6 and Table IA) and 236 genes (316 probes) that were downregulated in trMCF clone 11 (compared to MCF-10F) were upregulated by 10^−6^ M ATRA treatment (Fig. 6 and Table IB). These 443 genes defined a gene signature programming the reverse-transformation effect by ATRA (Table I). The relatively smaller number of significant probe sets in gene list-3 compared with other gene lists (Fig. 6) further supported the findings that ATRA-treatment reprograms the gene expression status of trMCF clone 11 cells to MCF-10F.

**Figure 7.**
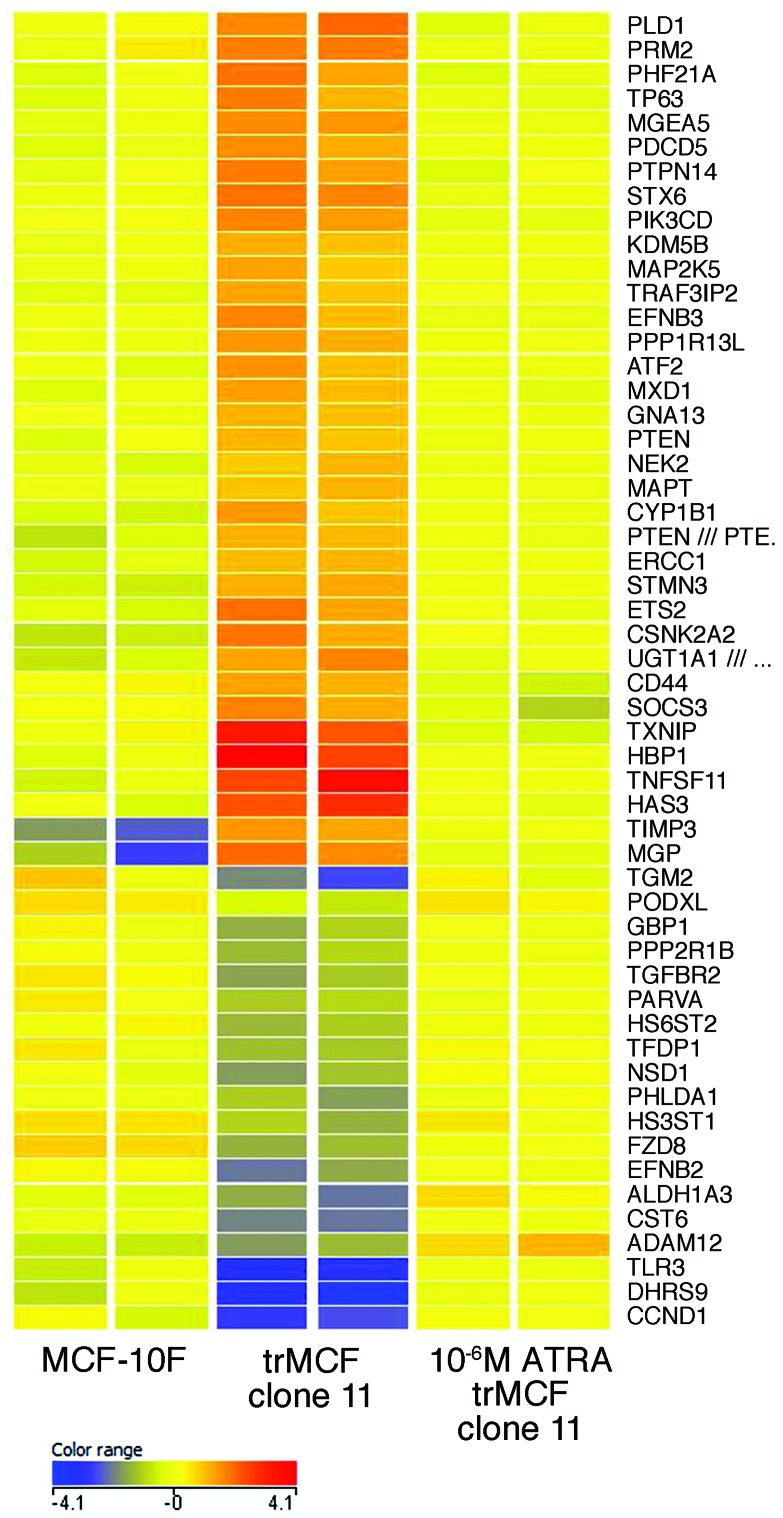
Heat map of selected genes in normal breast epithelial cells and early transformed cells before and after 10^−6^ M ATRA treatment. The expressions of genes involved in cell morphology are shown; also some genes from the aryl hydrocarbon and RAR pathways and genes involved in chromatin modification are shown. The genes that were dysregulated in the early transformed breast epithelial cells (trMCF clone 11) reached normal levels, similar to the normal breast epithelial cells MCF-10F, after treatment with 10^−6^ M ATRA. Red, yellow or blue colors represent expression levels above, at or below the mean level across the samples.

Ingenuity pathway analysis (IPA) revealed 4 canonical pathways significantly dysregulated in the transformed cells trMCF clone 11: aryl hydrocarbon receptor signaling, retinoic acid activation, xenobiotic metabolism signaling and molecular mechanism of cancer (Table II). Several genes of these pathways that were up- or downregulated in trMCF clone 11 show similar levels of expression to MCF-10F after trMCF clone 11 was treated with 10^−6^ M ATRA (Table II and Fig. 7). Genes from the aryl hydrocarbon receptor signaling ALDH1A3, CCND1, TGFBR2, TGM2 and TFDP1 were downregulated in the transformed cells trMCF clone 11 when compared to their expression in the normal breast epithelial cells MCF-10F and, the expression of these genes was upregulated after these cells were treated with 10^−6^ M ATRA reaching similar levels to the expression in MCF-10F (Table II and Fig. 7). One of the functions that show enrichment of dysregulated genes in the transformed trMCF clone 11 cells is cell morphology and the expression of most of these genes reached similar levels to MCF-10F after trMCF cells were treated with 10^−6^ M ATRA. The expression of some genes related to cell morphology such as PLD1, CD44, STX6, STMN3, ATF2, ETS2, NEK2, HAS3, MGP, GNA13 were upregulated in the transformed trMCF clone 11 and their expression reached normal levels after 10^−6^ M ATRA treatment (Fig. 7); other genes related to cell morphology such as PHLDA1, GBP1, HS6ST2 and TLR3 were downregulated in trMCF clone 11 and their expressions increased after 10^−6^ M ATRA treatment reaching similar levels to those found in the normal MCF-10F breast epithelial cells (Fig. 7). Also, the expression of several genes that encode enzymes involved in chromatin modifications such as MGEA5, ATF-2, KDM5B, PRMT2 (PRM2), PHF21A and NSD1, were dysregulated in trMCF clone 11, reaching normal levels after 10^−6^ M ATRA treatment (Fig. 7).

## Discussion

In this study we showed that all trans-retinoic acid (ATRA) induced branching of early transformed human breast epithelial cells. The transformed trMCF clone 11 cells form spherical masses in collagen (3D culture) and treatment with 10^−6^ M ATRA produced a significant decrease in spherical masses and an increased number of tubules. Cells at an advanced stage of transformation (bsMCF and caMCF) did not show any change in morphology after being treated with ATRA. Our previous results showed that RARβ (retinoic acid receptor β) was unmethylated in MCF-10F and trMCF cells and became hypermethylated at the invasive (bsMCF) and tumorigenic (caMCF) stages (19); although bsMCF and caMCF were treated with 5-aza-dC to reactivate the expression of RARβ in combination with ATRA, no changes in the phenotype of these cells in collagen were observed. Our results indicate that ATRA is able to re-differentiate early transformed cells to a normal stage but, not tumor cells at later stages of the neoplastic process. We previously showed that bsMCF and caMCF had important chromosomal gains and losses and the earlier transformed cells trMCF showed small genomic changes (16); this could explain why ATRA was only effective as a re-differentiation agent in the early transformed breast epithelial cells. Different studies indicate that epigenetic modifications play important roles in RA transcriptional regulation (20–24). Histones have a long N-terminal tail extending outside the nucleosome that is subject to acetylation, phosphorylation, and methylation (25). In the absence of RA, co-repressive elements (SMRT, NCoR and SIN3A) inhibit transcription; the presence of RA releases co-repressors and histone deacetylases allowing chromatin remodeling and access to specific RAREs (20,24). RA treatment leads to acetylation of histones H3 and H4 that lead to a more open stage of the chromatin allowing the transcription of ATRA regulated genes. However, only a limited number of information is currently available on the epigenetic dynamics of RA response.

Recently, analysis of gene expression array datasets of different FDA approved drugs revealed that ATRA (tretinoin) is a drug that is negatively associated with cancer stem cell (CSC) enriched gene expression signature (26). We found that ATRA treatment reduced the expression of the stem cell marker CD44 in early transformed cells. ATRA exerts effects on stem cell differentiation in part via the modulation of the epigenome. Numerous enzymes that alter the modifications on histones are involved in transcriptional activation of specific genes in stem cells, and many of these enzymes are modulated by RA treatment of stem cells (27). The expression of several genes encoding enzymes involved in chromatin modifications such as MGEA5, ATF-2, KDM5B, PRMT2, PHF21A and NSD1 were dysregulated in trMCF clone 11, reaching normal levels after ATRA treatment. Others have shown that in breast cancer, retinoids are effective inhibitors of breast cancer cells at early stages of tumor progression, but their effectiveness diminishes as the tumors become more aggressive (28). Our results support these findings.

Our results show that the RA concentration is important to induced re-differentiation of early transformed breast epithelial cells. The treatment of transformed cells with either 10^−7^ or 10^−8^ M ATRA did not induced any change in morphology although, cells were able to form tubules after treatment with 10^−5^ and 10^−6^ M ATRA, more tubules being developed after treatment with 10^−6^ M (1 *μ*M) ATRA.

Little is known about the genomic targets and effects of the different isoforms of the RARs and mechanism or extent of crosstalk between RA signaling and other signaling pathways. It has been recently shown that RAR binding through the genome is highly coincident with estrogen receptor α binding, resulting in widespread crosstalk of RA and estrogen signaling to antagonistically regulate breast-cancer associated genes (29). Our gene expression studies determined 443 genes which defined a signature of ATRA re-programming effect in early transformed breast epithelial cells; these genes were dysregulated in the early transformed cells and they reached normal levels after the cells were treated with 10^−6^ M ATRA. Genes from the aryl hydrocarbon receptor (AhR), retinoic acid receptor (RAR) and the xenobiotic pathways were dysregulated in the early transformed breast epithelial cells and their expression reached normal levels after ATRA treatment. It has been shown that there is an interaction between AhR and RAR activation and that AhR not only binds to polycyclic aromatic hydrocarbon family of environmental contaminants but also to some synthetic retinoids (30,31).

N-(4-hydoxyphenyl) retinamide (fenretinide or 4HPR) is a synthetic retinoid that is currently one of the most promising clinically tested retinoids. The modification of the carboxyl end of all-trans RA with N-4-hydroxyphenyl group resulted in increased efficacy as a chemoprevention agent as well as reduced toxicity when compared with other retinoids (32). Animal models have demonstrated that treatment with fenretinide prevents chemically induced cancers of the breast, prostate, bladder and skin (33–36).

In conclusion, our results showed that 1 *μ*M ATRA was able to re-differentiate transformed cells at early stages of the neoplastic process and antagonistically regulated breast cancer associated genes. Our results support previous findings that 1 *μ*M ATRA could be used as a chemo-preventive agent to inhibit the progression of premalignant lesions of the breast.

## Figures and Tables

**Table:** A, ATRA-downregulated genes (207 genes).

ACSS3	DNAJB9	KLF11	PL-5283/SLC13A4	TIMP3^a^
ALDH3A2	DSC3	KLHDC8B	PLAG1	TMEM167B
ALDOC	DUSP5P	LCA5	PLD1	TMEM27
ALPK1	EFHC1	LOC100288092	PLD6	TMEM40
ANKRD37	EFNB3	LOC100289187	PLK1S1	TMEM59
AQPEP	EPB41L4B	LOC100505894	POFUT2	TNFRSF25
ARG2	ERCC1^a^	LOC100506057/STK32C	POLR1D	TNFSF11
ARHGAP19	ETS2^a^	LOC100507303	PPIL6	TNKS
ARHGEF10	FABP6	LOC100507547	PPOX	TP63
ATF2	FAM117A	LOC100507644	PPP1R13L	TPD52L1^a^
ATG14	FAM168A	LOC439938	PPP1R3C	TPRG1
ATP2C2	FAM46C	LOC642587	PRKAB2	TRAF3IP2
ATP5C1	FAT2	LOX	PRMT2	TRAPPC6A
BCAS4	FBXO2	LRIG1	PROCR	TSC22D3
BFSP1	FEM1B	LYST	PTEN	TTBK2
BLNK	FLCN	MAP2K5	PTEN/PTENP1	TTC39B
BTBD3	FLJ37644	MAPT	PTPN14	TXNIP
C11orf80	FLJ45244	MGEA5	RAB11FIP4	UFM1
C16orf46	FNBP1L	MGP^a^	RAB38	UGT1A1/1A4/1A6/1A8/1A9/1A10
C17orf39	FNTA	MLF1	RAB40C	USP3
C17orf48	FNTB	MRAP2	RAB4A	USP32
C1orf133	FSIP1	MXD1	RAB7L1	VPS8
C1orf161	FXYD2	MYLIP	RASSF6	WAC
C20orf111	GBAS	N4BP2L1	RMND1	WDR59
C21orf7	GGNBP2	NDE1	RNF169	WDR91
C5orf41	GGTA1	NDUFB4	SCARA3	WWOX^a^
C7orf68	GIT2	NEK2	SCRG1	YOD1
C9orf9	GJA3	NEURL1B	SEMA6A	ZBTB34
CCDC28A	GKAP1	NFKBIL1	SFT2D1	ZFAND5
CD44^a^	GNA13	NGLY1	SHOX2	ZNF836
CELSR2	GNAI1	NMNAT3	SLC25A37	ZNRF1
CLCA2	GOSR2	NPL	SLC2A12	
CMBL	GPM6A	OGFRL1	SLC2A9	
COBL	GPNMB	PALMD	SLC5A3	
CRIP2	H19	PDCD4^a^	SOCS3	
CSNK2A2	HAS3	PDCD5	SORL1	
CYP1B1	HBP1	PDE7A	SPATA17	
CYP39A1	HERPUD1	PDZD2	STAU2	
DBP	HMGCL	PER1	STMN3	
DCD	IFNAR1	PER3	STX6	
DDAH2	IRF6	PHF21A	SUSD4	
DDC^a^	IRX2	PHLDB3	TESK2	
DDIT3	KCMF1	PHTF2	THBS2	
DHX40	KDM5B	PIK3CD	THSD1///THSD1P1	

Genes upregulated in trMCF clone 11 that were downregulated after 10^−6^ M ATRA-treatment to similar levels found in MCF-10F are indicated.

a Genes with binding sites for RARα or RARβ described by Hua *et al* (29).

**Table:** B, ATRA-upregulated genes (236 genes).

ABHD13	COX7B	HIATL1	OSTM1	SLC43A3	TSPAN2
ACP2	CRELD2	HIGD1A	P2RY2	SMPDL3A	UBE2N^a^
ADAM12	CST6^a^	HOXA11	PAPPA	SNRNP25	UBE2Q1
ALDH1A3	CSTF2	HPGD	PARVA	SNX19	UBP1
ANO1	CYB561D2	HS3ST1	PCSK5	SOAT1	UNK
AOX1	DCBLD2	HS6ST2	PDE12	SPAG1	VARS2
APOL6	DHRS9	IFI44	PHACTR3	SPATA13	VGLL3
ARGLU1	DHX9	IFIT3	PHLDA1	SRPX2	VSIG10
ARHGAP26	DNAJA1	IFIT5	PHLDA2	SRSF10	ZADH2
ARHGAP42	DOLK	IFNAR1	PITPNC1	SRSF2IP	ZBED4
ARHGDIB	DPH3	KHNYN	PKIB	SSPN	ZDHHC2
ARIH2	EFCAB2	KLHL18	PLCXD2	STK39	ZMPSTE24
ASPHD2	EFNB2	KLHL23	PLGLA/PLGLB1/PLGLB2	STS	ZNF252
ATP6V0A2	EHD4	KRT80	PNO1	STYK1	ZNF271
B3GALNT1	EIF2AK1	LOC100131993	PNPLA3	SUPT7L	ZNF326
BRI3BP	EIF5B	LOC100505759	PODXL	SUSD5	ZNF35
BTG1	ELOVL6	LOC100507192	POLR3K	SYNCRIP	
C12orf26	ENC1^a^	LOC283278	PPP2R1B	SYNJ2BP	
C12orf5	ENY2	LOC728903	PRPS1	SYTL2	
C1GALT1C1	ERLIN2	MACC1	PRR15	SYTL5	
C1orf116	EXOG	MARCKS	PSCA	TBC1D30	
C1orf135	FADS1^a^	MAT2A	PSME3	TFDP1	
C1orf212	FAIM	MCFD2	PTGR1	TFRC	
C1orf226	FAM118B	MEIS3P1	PTP4A2	TGFB2	
C6orf223	FAM119A	MFAP3L	PTPRB	TGFBR2^a^	
CALM1^a^	FAM83A	MFI2	PTPRJ	TGM2	
CCDC68	FBXW2	MFSD1	RABIF	THSD4	
CCDC88A	FDX1	MICALL1	RBM25	TIMM23	
CCND1^a^	FN1^a^	MMACHC	RBM45	TIMM8A	
CDA	FNIP2	MRPL35	RGS17	TIMM8B	
CDC42EP2	FRMD3	MST1R	RHOBTB1	TLCD1	
CDH2	FUCA1	MTERFD3	RHOF	TLR3	
CEP78	FXN	MYEOV	RPL27A	TLR4	
CFH/CFHR1	FZD8	MYO5C	RPS6KA2	TMC5	
CFI	GALNT7	NAA40	S1PR3	TMEM133	
CHAC2	GATAD2A	NAV3	SAMHD1	TMEM177	
CHML	GBP1	NECAP1	SCEL	TMEM9B	
CHRNA5	GDA	NIPAL1	SGK223	TP53I3	
CLDN23	GGCX	NMI	SH3TC2	TPCN2	
CMAH	GPATCH2	NRP2	SLC16A5	TRAK2	
CNPY2	GPX8	NSD1	SLC1A1	TRIM45	
COL4A3	GXYLT1	OLAH	SLC35B4	TRIOBP	
COL4A4	HAS2	OR7E14P	SLC35C1	TRNT1	
COX7A1	HERC6	OR7E47P	SLC37A1	TSPAN12	

Genes downregulated in trMCF clone 11 that were upregulated after 10^−6^ M ATRA-treatment to similar levels found in the normal breast epithelial cells MCF-10F.

a Genes with binding sites for RARα or RARβ described by Hua *et al* (29).

**Table II.** Canonical pathways enriched with differentially expressed genes.

	trMCF clone 11 vs. MCF-10F	ATRA trMCF clone 11 vs. trMCF clone 11
Aryl hydrocarbon receptor signaling	ALDH1A3↓, CCND1↓, TGFBR2↓, TGM2 ↓, TFDP1↓, ALDH3A2↑, CYP1B1↑	ALDH1A3↑, CCND1↑, TGFB2↑, TGM2↑,TFDP1↑, ALDH3A2↓, CYP1B1↓
	Other genes: CDKN1A↓, JUN↓ ALDH7A1↑, CSNK2A1↑, TGFB1↑, MAPK1↑, NFE2L2↑	Other genes: CCNE1↑, CCNE2↑, CDK6↑,DHFR↑, IL1B↑, IL6↑, NR2F1↑, NRIP1↑, POLA1↑ ALDH3B2↓, ARNT↓, NCOA3↓, HSPB2↓, ALDH6A1↓
RAR activation	ALDH1A3↓, DHRS9↓, NSD1↓, TGFB2↓, CSNK2A2↑, PIK3CD↑, PRMT2↑, PTEN↑	ALDH1A3↑, DHRS9↑, NSD1↑, TGFB2↑, CSNK2A2↓, PIK3CD↓, PRMT2↓, PTEN↓
	Other genes: JUN↓, NR2F2↓, RBP1↓, CSNK2A1↑, CSNK2A2↑, MAPK1↑, MAPK14↑, TGFB1↑, GNAS↑	Other genes: GTF2H2↑, IGFBP3↑, MAP2K1↑, MAPK13↑, NR2F1↑, NRIP1↑, RKAR2B↑, DH10↑, CITED2↓, PNRC1↓, PRKAR1A↓, SMARCD2↓
Xenobiotic metabolism signaling	ALDH1A3↓, HS3ST1↓, HS6ST2↓, PPP2R1B↓, ALDH3A2↑, CYP1B1↑, MAP2K5↑, PIK3CD↑, UGT1A1 (and others UGT)↑	ALDH1A3↑, HS3ST1↑, HS6ST2↑, PPP2R1B↑, ALDH3A2↓, CYP1B1↓, MAP2K5↓, PIK3CD↓, UGT1A1 (and others UGT)↓
	Other genes: CHST15↓, ALDH7A1↑, CAMK1D↑, HDAC4↑, MAPK1↑, MAPK14↑, MGMT↑, UGT8↑, NFE2L2↑	Other genes: ILIB↑, IL6↑, NRIP1↑, MAP2K1↑, MAPK13↑, ALDH3B2↓, ARNT↓, CAMK2D↓, CITED2↓, MAP3K8↓, PPP2R3A↓, ALDH6A1↓, MAP3K2↓
Molecular mechanisms of cancer	TGFB2↓, TGFBR2↓, CCND1↓, FZD8↓, PLCB4↓, RABIF↓, RHOF↓, GNA13↑, GNAI1↑, CD44↑	TGFB2↑, TGFBR2↑, CCND1↑, FZD8↑, PLCB4↑, RABIF↑, RHOF↑, GNA13↓, GNAI1↓, CD44↓
	Other genes: CDKN1A↓, CTNNB1↓, FYN↓, IRS1↓, JUN↓, SMAD4↓, TCF4↓, XIAP↓, PIK3CD↑, TCF3↑, TGFB1↑, GNAL↑, MAPK1↑, MAPK14↑	Other genes: APC↑, CCNE1↑, CCNE2↑, CDC25A↑, CDK6↑, CYCS↑, E2F2↑, MAP2K1↑, MAPK13↑, PRKAR2B↑, RAPGEF3↑, RBL1↑, TFDP1↑, ARHGEF10↓, FOXO1↓, HHAT↓, IRS1↓, NF1↓, PAK3↓, PIK3CD↓, PRKAR1A↓, RALGDS↓, RHOV↓

Genes downregulated (↓) or upregulated (↑) are shown. ATRA trMCF clone 11 refers to trMCF clone 11 treated with 10^−6^ M ATRA.
